# A Blood-Based Screening Tool for Alzheimer's Disease That Spans Serum and Plasma: Findings from TARC and ADNI

**DOI:** 10.1371/journal.pone.0028092

**Published:** 2011-12-07

**Authors:** Sid E. O'Bryant, Guanghua Xiao, Robert Barber, Ryan Huebinger, Kirk Wilhelmsen, Melissa Edwards, Neill Graff-Radford, Rachelle Doody, Ramon Diaz-Arrastia, for the Texas Alzheimer's Research & Care Consortium, for the Alzheimer's Disease Neuroimaging Initiative

**Affiliations:** 1 Department of Neurology, F. Marie Hall Institute for Rural and Community Health, Garrison Institute on Aging, Texas Tech University Health Sciences Center, Lubbock, Texas, United States of America; 2 Department of Clinical Sciences, University of Texas Southwestern Medical Center, Dallas, Texas, United States of America; 3 Department of Pharmacology and Neuroscience, Institute for Aging and Alzheimer's Disease Research, University of North Texas Health Science Center, Fort Worth, Texas, United States of America; 4 Department of Surgery, University of Texas Southwestern Medical Center, Dallas, Texas, United States of America; 5 Department of Genetics, University of North Carolina School of Medicine, Chapel Hill, North Carolina, United States of America; 6 Department of Psychology, Texas Tech University, Lubbock, Texas, United States of America; 7 Department of Neurology, Mayo Clinic, Jacksonville, Florida, United States of America; 8 Department of Neurology, Alzheimer's Disease and Memory Disorders Center, Baylor College of Medicine, Houston, Texas, United States of America; 9 Center for Neuroscience and Regenerative Medicine, Uniformed Services University of the Health Sciences, Rockville, Maryland, United States of America; Mental Health Research Institute of Victoria, Australia

## Abstract

**Context:**

There is no rapid and cost effective tool that can be implemented as a front-line screening tool for Alzheimer's disease (AD) at the population level.

**Objective:**

To generate and cross-validate a blood-based screener for AD that yields acceptable accuracy across both serum and plasma.

**Design, Setting, Participants:**

Analysis of serum biomarker proteins were conducted on 197 Alzheimer's disease (AD) participants and 199 control participants from the Texas Alzheimer's Research Consortium (TARC) with further analysis conducted on plasma proteins from 112 AD and 52 control participants from the Alzheimer's Disease Neuroimaging Initiative (ADNI). The full algorithm was derived from a biomarker risk score, clinical lab (glucose, triglycerides, total cholesterol, homocysteine), and demographic (age, gender, education, APOE*E4 status) data.

**Major Outcome Measures:**

Alzheimer's disease.

**Results:**

11 proteins met our criteria and were utilized for the biomarker risk score. The random forest (RF) biomarker risk score from the TARC serum samples (training set) yielded adequate accuracy in the ADNI plasma sample (training set) (AUC = 0.70, sensitivity (SN) = 0.54 and specificity (SP) = 0.78), which was below that obtained from ADNI cerebral spinal fluid (CSF) analyses (t-tau/Aβ ratio AUC = 0.92). However, the full algorithm yielded excellent accuracy (AUC = 0.88, SN = 0.75, and SP = 0.91). The likelihood ratio of having AD based on a positive test finding (LR+) = 7.03 (SE = 1.17; 95% CI = 4.49–14.47), the likelihood ratio of not having AD based on the algorithm (LR−) = 3.55 (SE = 1.15; 2.22–5.71), and the odds ratio of AD were calculated in the ADNI cohort (OR) = 28.70 (1.55; 95% CI = 11.86–69.47).

**Conclusions:**

It is possible to create a blood-based screening algorithm that works across both serum and plasma that provides a comparable screening accuracy to that obtained from CSF analyses.

## Introduction

Alzheimer's disease (AD) is a devastating disease affecting millions of people worldwide. While a Food and Drug Administration (FDA) working group recently provided preliminary approval for a beta amyloid (Aβ) neuroimaging technique as a biological marker (Amyvid^©^, Elli Lilly), no blood-based biomarker screening tool has received approval to date. However, blood-based biomarkers present significant advantages over neuroimaging modalities. For example, blood-based screenings offer a cost effective method of screening candidates for therapeutic trials [1], provide a rapid, cost-effective means of screening for AD at the population level [2], [3], [4], [5], and provide an optimal starting point for a multi-stage assessment process that can be followed-up by clinical modalities (i.e. medical exam, neuropsychological testing, standard neuroimaging, clinical bloodwork), specialized neuroimaging (i.e. Aβ imaging, fMRI, volumetric MRI analyses), and/or CSF (i.e. t-tau, Aβ_1–42_, and/or t-tau/Aβ_1–42_ ratio score) analyses [4] for screen positive cases. The 2009 U.S. Census estimates suggested that there were nearly 40 million Americans age 65 and above with an additional 34 million reaching 65 within 10 years; there are many more world-wide. Given their cost and limited availability, available imaging, clinical, and CSF modalities are not reasonable first-line approaches for screening all elders at risk of having AD or that have concerns about having the disease. The purpose of this study was to generate and cross-validate a blood-based screener for AD that can be incorporated into the existing medical infrastructure with additional assessments (e.g. clinical, imaging, CSF analysis) to confirm those who screen positive.

In the last several years, there have been significant advancements in the search for blood-based biomarkers for Alzheimer's disease (AD). In 2007, Ray and colleagues [6] analyzed a panel of plasma-based proteins among samples from 259 controls, AD and mild cognitive impairment (MCI) cases and generated a biomarker algorithm that accurately identified 89% of those with and without the disease; however, this work has not been replicated [7]. Buerger and colleagues [8] examined blood-based microcirculation markers as possible diagnostic markers for AD (AD n = 94, controls n = 53). These authors found that a ratio score of pro-atrial natriuretic peptide (MR-proANP) to C-terminal endothelin-1 precursor fragment (CT-proET-1)(MR-proANP/CT-proET-1 ratio) from plasma yielded a sensitivity of 0.81 and specificity of 0.82 in discriminating probable AD from healthy controls. More recently, we created a biomarker risk score from serum proteins (AD n = 197, controls n = 203) that yielded a 91% overall accuracy [2]. Our approach took the algorithm a step further by combining both demographic (i.e. age, gender, education, and APOE*E4 status) and clinical lab values (i.e. cholesterol, triglycerides, high density lipoproteins, low density lipoproteins, lipoprotein-associated phospholipase, homocysteine, and C-peptide) into the algorithm, which improved the overall accuracy to 95% [5]. Analyzing samples from 22 AD cases, 22 controls, and 12 non-AD disease comparison subjects, Reddy and colleagues [9] took a novel approach by examining serum IgG antibodies as potential biomarkers of AD status obtaining impressive results (AUC = 0.99); however, the sample size was very small (n = 15 AD cases in test set) limiting the generalizability of the findings at this point. Together, these studies suggest that a blood-based screening tool for AD is on the horizon.

Although this work is promising, there is little consistency as to what biological fluid is used for biomarker assays (i.e. serum versus plasma), which may explain many inconsistent findings found in the literature. While some assays must be conducted in one medium or another, there are numerous studies linking a variety of blood-based markers to AD from both mediums. Mayeux and colleagues [10] analyzed *plasma* amyloid β (Aβ) peptides Aβ_1–40_ and Aβ_1–42_ on 530 participants and found that Aβ_1–42_ (but not Aβ_1–40_) levels were higher among baseline AD cases as well as those who developed AD over a three-year period as compared to those who did not. Luis et al. [11] analyzed *serum* Aβ_1–40_ and Aβ_1–42_ levels among a sample of 87 AD and MCI cases as well as controls. In that study, serum Aβ_1–40_ levels did not differ between groups whereas serum Aβ_1–42_ levels where highest among MCI cases (versus AD cases and controls) and controls and AD levels were intermediate between those of the MCI cases and controls. The *serum* Aβ_1–42/1–40_ ratio was also highest among the MCI group. In a sample of 40 AD cases and controls, Laske et al. [12] found that *serum* brain derived neurotrophic factor (BDNF) levels varied according to AD severity, suggesting BDNF as a potential biomarker for AD, though we failed to cross-validate these findings in a sample of 198 AD cases and controls from the Texas Alzheimer's Research Consortium (TARC) cohort [13]. In a follow-up study of 399 AD cases and controls, *elevated* serum BDNF was found to be specifically related to poorer memory performance among AD cases [14] whereas Komulainen and colleagues [15] found that *lower plasma* BDNF levels were significantly related to poorer scores on tests of language and memory among women in a population based sample of aging men and women (n = 1389).

To date, we are aware of no prior work that has explicitly sought to find blood-based biomarkers of AD across both serum and plasma and with no previous attempts at identifying blood-based screening tools utilizing markers across blood fractions. Additionally, no previously created blood-based tools have been cross-validated in independent cohorts. The current study was designed to (1) identify blood-based proteins that were highly correlated across both serum and plasma that also were significantly related to AD status, and (2) generate a screening algorithm for AD utilizing those markers from serum in the TARC cohort and validate that algorithm in the Alzheimer's Disease Neuriomaging Initiative (ADNI) plasma-samples. We hypothesized that, as with our prior work, we would be able to generate a screening algorithm that accurately identified AD across cohorts.

## Methods

### Participants

Texas Alzheimer's Research Consortium (TARC). Serum protein data were analyzed from 396 participants (197 AD subjects, 199 controls) from the TARC longitudinal cohort. In addition, plasma protein data were analyzed on a matched sample of 40 AD cases from the TARC. Blood samples for comparison of plasma and serum proteins were drawn concurrently from the same individuals. The methodology of the TARC project has been described in detail elsewhere [2], [16]. Briefly, each participant undergoes a standardized annual examination at the respective sites, which includes a medical evaluation, neuropsychological testing, interview, and blood draw for storage of samples in the TARC biobank. Diagnosis of AD was based on NINCDS-ADRDA criteria [17] utilizing consensus review. Institution Review Board approval was obtained for this study with each participant (or caregiver) providing written informed consent. The Institution Review Board (IRB) at Texas Tech University Health Sciences Center, Baylor College of Medicine, University of North Texas Health Science Center, the University of Texas Southwestern Medical Center, and the University of Texas Health Science Center - San Antonio approved this research.

Alzheimer's Disease Neuroimaging Initiative (ADNI). Data used in the preparation of this article were obtained from the ADNI database (adni.loni.ucla.edu). The ADNI was launched in 2003 by the National Institute on Aging (NIA), the National Institute of Biomedical Imaging and Bioengineering (NIBIB), the Food and Drug Administration (FDA), private pharmaceutical companies and non-profit organizations, as a $60 million, 5-year public-private partnership. The primary goal of ADNI has been to test whether serial magnetic resonance imaging (MRI), positron emission tomography (PET), other biological markers, and clinical and neuropsychological assessment can be combined to measure the progression of mild cognitive impairment (MCI) and early Alzheimer's disease (AD). The Principal Investigator of this initiative is Michael W. Weiner, MD, VA Medical Center and University of California – San Francisco. ADNI is the result of efforts of many co-investigators from a broad range of academic institutions and private corporations, and subjects have been recruited from over 50 sites across the U.S. and Canada. For up-to-date information, see www.adni-info.org. Data from 170 participants from ADNI (58 controls and 112 AD cases) for whom plasma-based protein results were available were utilized in this study.

Blood Assays. In TARC, non-fasting samples were collected whereas ADNI utilized a fasting blood collection procedure. Serum blood samples were collected in serum-separating tubes during clinical evaluations, allowed to clot at room temperature for 30 minutes, centrifuged, aliquoted, and stored in polypropylene tubes at −80°C. In both TARC and ADNI, plasma samples were collected in lavender-top tubes and gently mixed 10–12 times. Next tubes were centrifuged at room temperature and plasma extracted and frozen until assay. In both studies, serum and plasma samples were sent to Rules Based Medicine (RBM, www.rulesbasedmedicine.com, Austin, TX) for assay on the RBM multiplexed immunoassay human Multi-Analyte Profile (humanMAP). Individual proteins were quantified with immunoassays on colored microspheres. Information regarding the least detectable dose (LDD), inter-run coefficient of variation, dynamic range, overall spiked standard recovery, and cross-reactivity with other humanMAP analytes can be readily obtained from RBM. Clinical lab data. Homocysteine, hemoglobin A1c, c-peptide, and lipoprotein-associated phospholipase A2 (Lp-PLA2) was provided by the Ballantyne laboratory at Baylor College of Medicine. Sample collection and storage was as described above. Lipids were measured using a AU400e automated chemistry analyzer (Olympus America; Center Valley, PA), serum total homocysteine (tHcy) by recombinant enzymatic cycling assay (Roche Hitachi 911), c-peptide by enzyme-linked immunosorbent assay (ELISA), HbA1c measurement by turbidimetric inhibition immunoassay (TINIA) for hemolyzed whole blood and Lp-PLA2 levels by diaDexus PLAC® test (diaDexus, Inc, San Francisco, CA). Clinical lab data from ADNI was conducted using kits provided by Covance. ADNI CSF Biomarkers. Our blood-based algorithm was compared to the diagnostic accuracy of the total tau (t-tau) to beta amyloid (Aβ_1–42_) ratio (t-tau/Aβ_1–42_) previously completed as part of the ADNI protocol. The CSF methods for ADNI have been described in detail elsewhere [18]. Lumbar punctures were conducted with a median of one day after baseline clinical visit. Once CSF was transferred into polypropylene tubes it was frozen and shipped to the ADNI Biomarker Core laboratory at the University of Pennsylvania Medical Center where biomarker assays were conducted [18].

**Figure 1:**
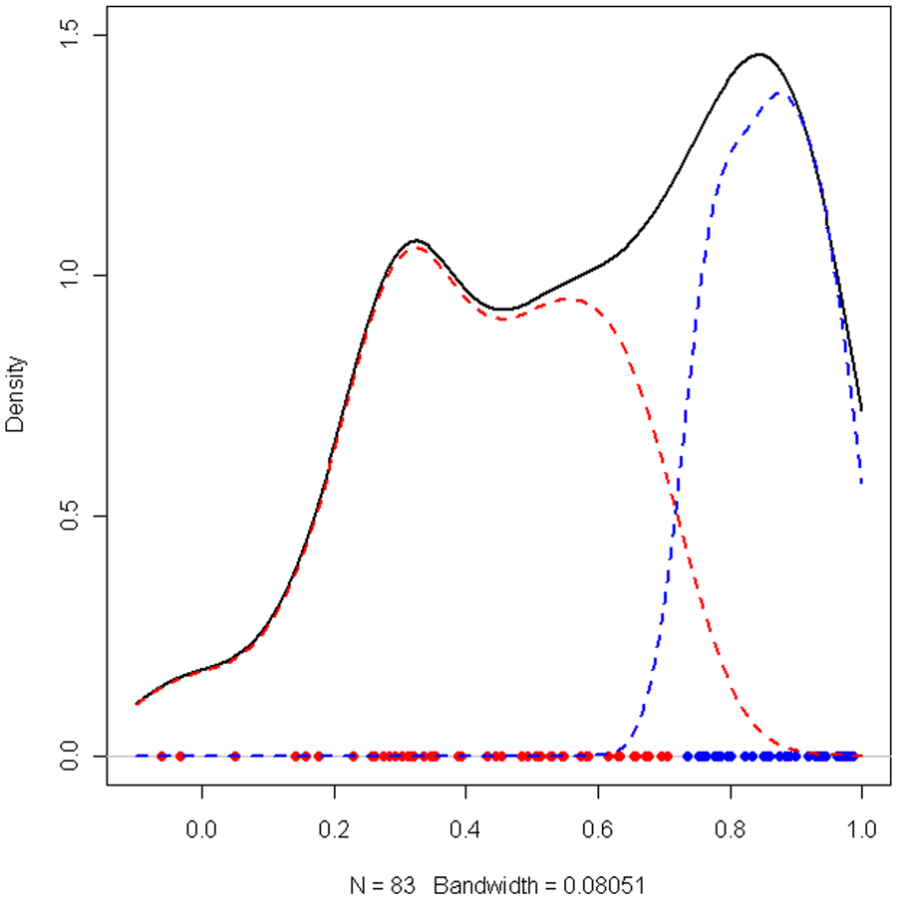
The density plot the Pearson's correlation coefficients between serum and plasma in TARC cohort. We used Mclust (model-based clustering algorithm [21]) package in R to fit the data and discovered two clusters in the correlation coefficients: one (red) corresponding to low correlation and the other (blue) corresponding to high correlation. The threshold value that separated these two clusters most effectively is 0.75. The black line is the density plot of all biomarkers. The dots represent the correlation coefficients of the biomarkers and the color indicates the cluster membership.

**Figure 2:**
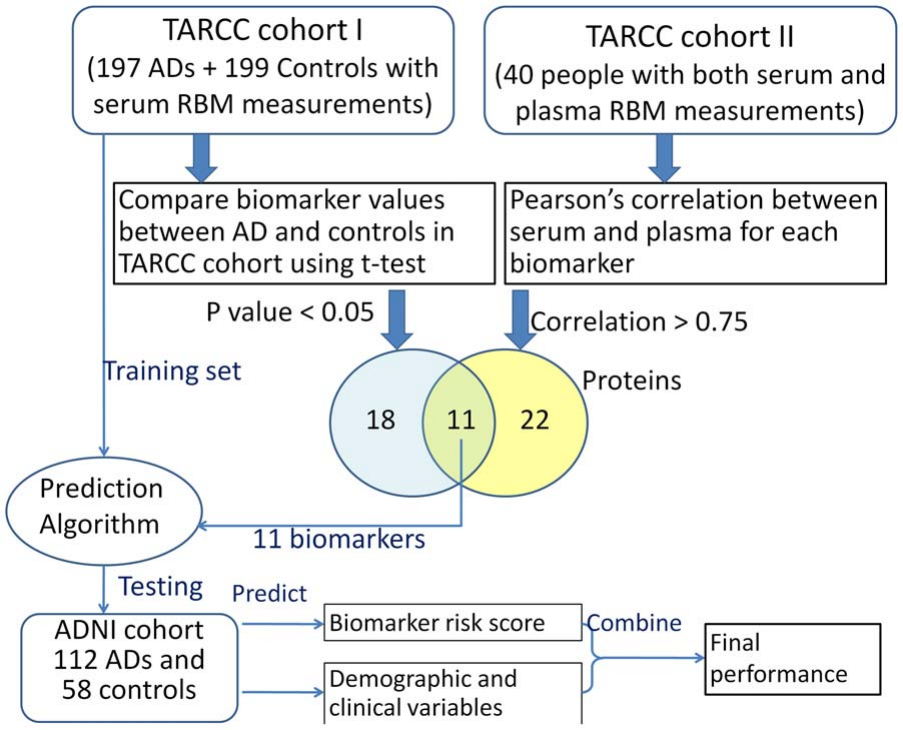
Outline of methods.

Statistical Analyses. Analyses were performed using R (V 2.10) statistical software [19]. Biomarker data were transformed using Box-Cox [20] transformation so that the distribution of each protein is approximately normal. Analyses took place in a series of steps. Identification of proteins across serum and plasma. Pearson correlations were conducted in the TARC sub-sample across serum and plasma proteins to determine which markers were comparable across mediums. Model-based clustering algorithm [21] (Mclust package in R) was used to empirically determine the optimal correlation cut-off that separated the highly correlated versus weakly correlated proteins. The optimal cut-score was 0.75, which identified 33 proteins with high correlation (≥0.75) between serum and plasma (see Figure 1). T-test analyses comparing the abundance of proteins between AD and controls identified 29 that were differentially expressed between groups (p<0.05) in full the TARC cohort (training set). Eleven proteins were significantly different between AD and control participants and were found to be correlated ≥0.75 across serum and plasma. These 11 proteins are defined as protein biomarkers in this study. Figure 2 reflects a graphic representation of the methods. Development of Biomarker Diagnostic Model. Next, we used the 11 protein biomarkers to develop our prediction model using random forest (RF) method [22], [23], implemented using R package *randomforest* (V 4.5) [22]. The TARC cohort was designated as the training sample in which the prediction model was derived. Validation of the Prediction Model. The protein biomarker-based RF prediction model derived from the TARC serum-based biomarker training set (TARC) was applied to the ADNI plasma-based dataset (test sample) to predict the risk score for each patient in the ADNI cohort. Of note, no ADNI data were utilized in (1) identification of serum-plasma comparable proteins or (2) development of the RF prediction model. This was done to avoid the overfitting or other possible confounds across medium and/or cohorts. Diagnostic Accuracy. Diagnostic accuracy was evaluated by examining the area under the receiver operating characteristic (ROC) curves (AUC). Our approach to creating a blood-based diagnostic algorithm for AD is to combine the predicted biomarker risk score from the RF model with demographic and clinical lab data via a multivariate logistic regression model. Demographic data incorporated into the algorithm was age, gender, level of education, and presence of APOE*E4 genotype (homozygous or heterozygous) while clinical lab data included glucose, triglycerides, total cholesterol, and homocysteine. These variables were included as they were (1) available from both cohorts and (2) have been linked to AD. Lastly, the likelihood ratios of having AD based on a positive test finding (LR+), the likelihood ratio of not having AD based on the algorithm (LR-) and the odds ratio of AD were calculated in the ADNI cohort.

## Results

Demographic characteristics of the samples are provided in Table 1. Eleven proteins met our criteria of (1) having a correlation coefficient ≥0.75 between serum and plasma in the same participant and (2) being associated with disease status p<0.05. The 11 proteins were as follows: C-reactive protein, adiponectin, pancreatic polypeptide, fatty acid binding protein, interleukin 18, beta 2 microglobulin, tenascin C, T lymphocyte secreted protein 1.309, factor VII, vascular cell adhesion molecule 1, and monocyte chemotactic protein 1. See Table 2 for correlations among serum and plasma for these 11 proteins as well as the mean differences between cases and control groups of these biomarkers and clinical lab data across cohorts.

**Table 1:** Demographic characteristics of the cohorts.

	TARC – serum sample			TARC – plasma sample	ADNI		
	AD (N = 197)	Control (N = 198)	p-value	AD (n = 40)	AD (n = 112)	Control (n = 58)	p-value
Gender (male)	34.5%	31.3%	0.52	40%	42%	48%	0.52
Age (years, mean/sd)	77.4(8.3)	70.4(8.9)	<0.001	75.7(1.6)	75.2(8.1)	75.5(5.8)	0.63
Education (years, mean/sd)	14.0(3.5)	15.5(2.7)	<0.001	14.5(0.6)	15.1(3.2)	15.6(2.7)	0.38
APOE*E4 positive	59.3%	26.5%	<0.001	50%	68%	9%	<0.001

Note: TARC = Texas Alzheimer's Research Consortium; ADNI = Alzheimer's Disease Neuroimaging Initiative. Fisher exact test was used for categorical outcomes (Gender, APOE*E4 positive) and Wilcoxon test was used for continuous outcomes (Age, Education).

**Table 2:** Biomarkers and Clinical Labs Across Cohorts.

Marker	Pearson correlation for serum vs. plasma (TARC cohort)	Mean difference in TARCC	Mean difference in ADNI
C Reactive Protein	0.97	−3.35	−2.07
Adiponectin	0.95	1.88	1.79
Pancreatic polypeptide	0.89	4.29	2.78
Fatty Acid Binding Protein	0.88	1.72	−0.79
IL 18	0.86	−1.87	0.51
Beta 2 Microglobulin	0.85	3.14	2.09
Tenascin C	0.85	4.56	2.93
I.309	0.8	1.12	−1.68
Factor VII	0.8	−2.78	−1.26
VCAM 1	0.78	3.00	2.82
MCP 1	0.75	−2.74	−0.30
Total Cholesterol	–	0.13	0.78
Triglycerides	–	−0.63	1.59
Homocysteine	–	3.99	1.06

Note: Mean difference reflects the mean difference between cases and controls divided by the its standard deviation.

**Figure 3:**
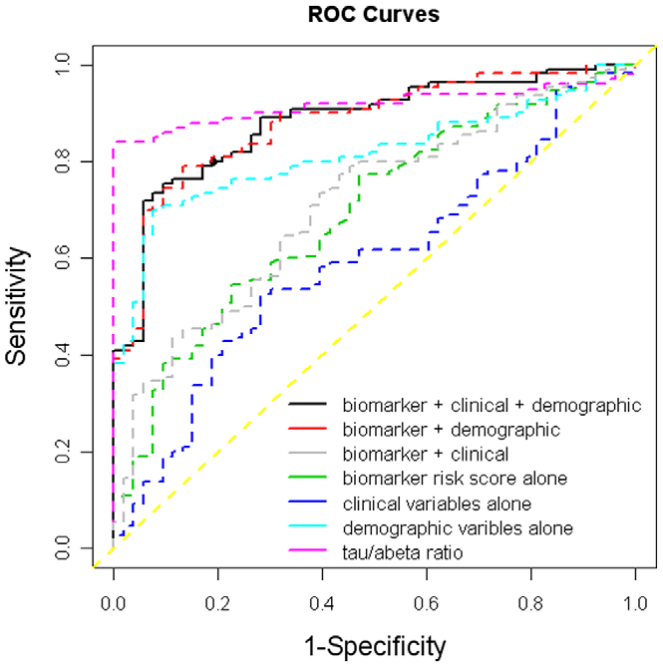
ROC curve for serum-plasma based biomarker algorithm. Each line represents the AUC of the respective portions of the algorithm with the yellow line reflecting chance.

The optimal cut-score for the RF biomarker risk score from the test sample (ADNI) was 0.51 which obtained AUC of 0.70 with a sensitivity (SN) and specificity (SP) of 0.54 and 0.78, respectively. For comparison purposes, the ADNI CSF t-tau/Aβ_1–42_ ratio yielded a superior diagnostic accuracy with an observed AUC = 0.92, SN = 0.84, and SP = 1.00. However, as with our prior approach, when the biomarker risk score was combined with demographic and clinical lab data [2], [5], the precision improved substantially. Our combined algorithm yielded a much better diagnostic accuracy with an observed AUC = 0.88, SN = 0.75, and SP = 0.91. Of note, the diagnostic accuracy of our serum-plasma based algorithm was comparable to that obtained from ADNI CSF analyses. See Table 3 and Figure 3. The likelihood ratio positive (LR+) was 7.03 (SE = 1.17; 95% CI = 4.49–14.47), the likelihood ratio negative (LR−) was 3.55 (SE = 1.15; 2.22–5.71), and the odds ratio (OR) was 28.70 (1.55; 95% CI = 11.86–69.47). The misclassification rate was 14% (95% CI = 9–21%). If we set SN at 0.80 for our full algorithm, the resulting SP was 0.81, which also meets the criteria for the Consensus Report of the Working Group on Molecular and Biochemical Markers of AD [24].

**Table 3:** Diagnostic accuracy of the serum-plasma algorithm.

	AUC (95% CI)	SN (95% CI)	SP (95% CI)
biomarker + clinical + demographic	0.88 (0.83–0.93)	0.75 (0.67–0.83)	0.91 (0.80–0.96)
biomarker + demographic	0.88 (0.83–0.93)	0.79 (0.71–0.86)	0.87 (0.75–0.93)
Biomarker + clinical	0.71 (0.63–0.79)	0.73 (0.64–0.81)	0.60 (0.47–0.72)
biomarker risk score alone	0.70 (0.62–0.78)	0.54 (0.45–0.63)	0.78 (0.65–0.87)
clinical variables alone	0.59 (0.50–0.68)	0.53 (0.43–0.62)	0.72 (0.58–0.82)
demographic variables alone	0.81 (0.75–0.88)	0.70 (0.61–0.78)	0.92 (0.82–0.97)
CSF tau/abeta ratio	0.92 (0.87–0.96)	0.84 (0.76–0.90)	1.00 (0.93–1.00)

Note: AUC = area under the receiver operating characteristic curve; SN = sensitivity; SP = specificity; CI = confidence interval; demographic = age, gender, education, APOE*E4 status (presence/absence); clinical = glucose, triglycerides, total cholesterol, homocysteine.

## Discussion

In the current study we demonstrate that (1) there are proteins that are highly correlated in plasma and serum and are associated with AD status across blood fractions, (2) these findings are replicable across independent cohorts, and (3) using these proteins, we generated a prediction model in the TARC cohort that, when combined with demographic and clinical lab data, yielded clinically significant classification accuracy in the ADNI cohort. To date, this is the first blood-based screener for AD developed that has been cross-validated in an independent large-scale cohort that also works across blood fractions. This work not only further supports the notion that an accurate blood-based screening tool for AD can be generated, but also that such an algorithm can be applied across serum and plasma mediums. Our 11-protein serum-plasma risk score alone yielded an AUC of 0.70 accuracy that was enhanced by the addition of demographic (i.e. age, gender, education, APOE*E4 status) and clinical lab (i.e. glucose, triglycerides, total cholesterol, and homocysteine) data. In Table 3, the addition of clinical lab data did not improve the overall accuracy of the algorithm beyond demographic information, which is largely driven by the APOE*E4 rates in the ADNI cohort. However, in our prior work [5], the use of clinical lab data improved overall accuracy and will likely contribute to the robustness of our approach as it is applied to other cohorts. It is certainly possible that inclusion of additional markers, not available in the current analyses, would increase the accuracy of that risk score, which is an additional advantage of our approach as it can be expanded or reduced as necessary to support the accuracy and cost-effectiveness of the algorithm. A single biomarker algorithm that works across both serum and plasma will offer laboratories options that may be preferable for a variety of reasons.

There are several implications for the current findings. There are a number of previously conducted research projects with stored blood biospecimens; however, there is little consistency between what medium was stored. The current findings open up the possibility of utilizing samples from such studies to further validate and refine our algorithm. Additionally, it is likely that the components of diagnostic algorithms will be different from the components of algorithms for progression and different from those predicting long-term risk. Our findings offer a novel approach to each of these questions as well. These findings also support the need for standard protocols to be generated for blood-based AD biomarker research as is currently underway for the CSF markers.

These results also support the robustness of our methodological approach. In our initial serum-based algorithm, the biomarker risk score alone yielded an AUC of 0.91 whereas the serum-plasma algorithm in the current study yielded an AUC of 0.70. While impressive, this overall accuracy is not clinically adequate. However, as with our prior approach, the combination of clinical lab data and demographic variables into the algorithm increased the precision substantially (AUC = 0.88). In our prior work, the training and test sample were both based on serum assays and were from the larger TARC cohort; however, the derivation of the algorithm in the TARC cohort and validation in the ADNI cohort supports the robustness of this method. As we have previously argued, using only age, gender, education and APOE*E4 status, one can accurately classify a large number of AD cases when compared to controls. Therefore, consideration of such factors should be considered when examining biomarkers of AD status. We are not the first to demonstrate that inclusion of these factors into an algorithm can improve overall accuracy as others have suggested that a multi-marker approach is superior to single-marker approaches [25], [26]. As an example, Vemuri and colleagues found that including demographic factors with structural MRI added to the overall accuracy of disease-prediction models even when cases and controls were matched by these variables [27]. This is important given that the TARC cohort did not match cases and controls whereas ADNI samples were matched. The robustness of our methodology may also provide an explanation for the lack of cross-validation of prior work [6], [7]. The utility of our algorithm for separating MCI cases from normal controls (and/or AD) remains unknown at present.

The current markers overlap with our prior serum-only based algorithm [2], [5] though they do not overlap with those found by Ray and colleagues [6], which may be due to the significant differences in assay platforms utilized. However, there is an existing literature directly or indirectly linking each of the 11 proteins identified in this study to AD. As with our prior work, many of the markers in the algorithm are inflammatory in nature, which we propose as evidence of an inflammatory endophenotype of AD [2], [28]. We, and others, have documented a link between CRP and AD [28]. Based on the available data, we proposed that the link between CRP and the risk of AD changes over the life course with midlife elevations in CRP increasing risk for AD, but that this risk declines as one ages with decreased CRP related to AD status though elevations in CRP are still related to increased disease severity among cases [28]. Adiponectin, an adipocytokine, is related to obesity, insulin resistance, metabolic syndrome, type 2 diabetes, and cardiovascular disease [29] and was recently found to be elevated in plasma among MCI and AD cases [30]. Therefore, adiponectin levels may be related to the documented links between changes in body composition (e.g. weight loss) seen in prodromal and early stage AD. Pancreatic polypeptide is also linked with diabetes and obesity [31], [32] and may provide a clue into the biological link between these conditions and AD. Fatty acid binding proteins, cytosolic proteins found in all cells utilizing fatty acids, are rapidly released into circulation following cell damage [33]. Serum levels fatty acid binding proteins have been shown to be elevated among AD and other dementia cases as compared to normal controls [33], [34]. A recent meta-analysis showed a significant up-regulation in blood concentrations of IL-18 (as well as IL-6, TNFα, IL1, transforming growth factor, IL-12) among AD cases [35]. β2 microglobulin is an amyloid protein [36] that has been found to be elevated in the CSF of AD cases [37], [38]. Tenascin-C, an extracellular matrix glycoprotein, is involved in a number of biological processes that have been linked to AD including inflammation and angiogenesis [39], which may provide a biological mechanism linking AD to a broad spectrum of cardiovascular diseases and risk factors. The human cytokine I-309, a small glycoprotein, was recently found to be elevated in a proteomic study of CSF among AD cases and was also related to scores on a test of global cognitive functioning (i.e. Mini Mental State Examination [MMSE]) [40]. Factor VII is a protein in the coagulation cascade that is required for thrombin generation, which has also been linked to AD [41]. VCAM-1 is a member of the immunoglobulin superfamily that has been found elevated in plasma of AD cases [42]. It has been proposed that MCP-1 plays a dominant role in the chronic inflammation seen in AD [43] and has been found to be elevated in serum of patients diagnosed with MCI and mild AD [44].

Given the sheer volume of elders worldwide who are at risk for AD, there is an urgent need for a multi-stage approach to screening and diagnosis. There are insufficient numbers of dementia experts to meet the needs of all individuals at risk for the disease and prior work has demonstrated that non-experts are not completely accurate in diagnosing the disease [45], particularly in the earlier stages [46]. Our blood-based screener fits into the existing medical infrastructure where screen positives can be referred for confirmatory diagnosis using clinical, imaging, and/or CSF analysis. As with any screening measure, one must consider acceptable levels of false positive and false negative rates of the instrument as well as overall disease base rates of the setting when deciding on appropriate cut-scores on any instrument [47]. Therefore it is important that additional work be conducted to determine how this algorithm (and other previously published biomarkers) performs in community-based settings (e.g. primary care offices) as both the TARC and ADNI are clinic-based cohort studies. While sensitivity and specificity are not base rate dependent, accuracy of diagnosis (prediction of disease status present/absent) is a function of base rates of the disease within a given population therefore, overall accuracy of AD presence (i.e. true positives) will increase with advancing age while accuracy of AD absence (i.e. true negatives) will be higher with younger ages. As with age, APOE*E4 genotype, gender, and/or years of education are also important considerations, which is why these variables are included in the algorithm itself.

The independent cohorts strongly support the validity of the findings. These observations also justify further analysis examining a broader range of markers across serum and plasma to determine if the biomarker risk score can be further refined. Our results also suggest that further work in the field should specifically examine the performance of blood-based protein panels across serum and plasma.
